# Outcomes among newly diagnosed AL amyloidosis patients with a very high NT-proBNP: implications for trial design

**DOI:** 10.1038/s41375-021-01297-z

**Published:** 2021-05-21

**Authors:** I. Vaxman, S. K. Kumar, F. Buadi, M. Q. Lacy, D. Dingli, Y. Hwa, A. Fonder, M. Hobbs, S. Hayman, T. Kourelis, R. Warsame, E. Muchtar, N. Leung, P. Kapoor, M. Grogan, R. Go, Y. Lin, W. Gonsalves, M. Siddiqui, R. A. Kyle, S. V. Rajkumar, M. A. Gertz, A. Dispenzieri

**Affiliations:** 1 Division of Hematology, Mayo Clinic, Rochester, MN USA; 2 Institute of Hematology, Davidoff Cancer Center, Rabin Medical Center, Petah Tikva, Israel; 3 Sackler Faculty of Medicine, Tel-Aviv University, Tel-Aviv, Israel; 4 Department of Cardiovascular Medicine, Mayo Clinic, Rochester, MN USA

**Keywords:** Risk factors, Myeloma

## To the Editor:

Prognosis in AL amyloidosis is variable and is a function of plasma cell biology (serum-free light chains, genetics, bone marrow plasma cell burden, depth of hematologic response to treatment and organ involvement) [1]. There are two commonly used prognostic scores for staging in AL amyloidosis [2–4], both use cardiac biomarkers to predict survival: European modification of Mayo 2004 and Mayo 2012, which uses slightly different cut-offs and adds the difference between involved and uninvolved light chain (dFLC) into the model.

It is well documented that overall survival (OS) in AL amyloidosis is improving over time, in part due to earlier diagnosis and in part due to more effective therapies [5, 6]. For the purposes of clinical trials, patients with the highest-risk disease are often excluded from trial participation [7, 8]. An N-terminal pro-brain natriuretic peptide (NT-proBNP) of 8500 ng/L or higher is the criterion most often used for exclusion from clinical trials due to high rates of early death [4, 8]. In an effort to set the stage for better clinical trial design, we aimed to characterize the outcomes of patients with very high (VH) NT-proBNP (≥8500 ng/L) and evaluate the prognostic scores in terms of their ability to stage discriminate patients with and without VH NT-proBNP.

We retrospectively identified newly diagnosed AL amyloidosis patients who were diagnosed between January 2012 and July 2020 (*n* = 1290). Patients were excluded if they were not seen at Mayo Clinic within 90 days of diagnosis (*n* = 291) and if they did not have baseline biomarkers (*n* = 170), leaving 829 patients for our analysis.

The diagnosis and staging of AL amyloidosis were according to consensus criteria [2–4, 9]. Thresholds for troponins and BNPs were corrected using a conversion method previously described by our group [10]. The vast majority of patients had troponin T measured, and for them, the 0.025 mcg/L and the 0.035 mcg/L cut-points were used for the 2012 and 2004 staging systems, respectively. A minority of patients did not have troponin T, but rather high sensitivity troponin T (*n* = 129) or troponin I (*n* = 23). For patients with high sensitivity troponin T, cut-points of 40 and 50 ng/L were used, respectively, for the 2012 and 2004 systems, and for patients with troponin I only, a cut-point of 0.1 mcg/L was used for both systems. In the three patients with no NT-proBNP but with BNP, 400 and 81 ng/L were used respectively for the 2012 and 2004 systems; otherwise, the 1800 ng/L and 332 ng/L cut-offs were used for NT-proBNP. A BNP of >700 ng/L was considered equivalent to NT-proBNP ≥8500 ng/L. VH NT-proBNP was defined as NT-proBNP ≥8500 ng/L or equivalent.

First-line treatments were chosen by treating physicians based on the extent of cardiac involvement, age, performance status, drug availability and patient’s preference. Organ involvement was defined according to existing criteria [9]. Patients underwent ASCT if they were eligible according to the mSMART criteria. [1]

Patient and disease factors were compared for categorical and continuous variables using (*χ*^2^, or Fisher’s exact) and (*t*-test, or Wilcoxon signed-rank test), respectively. OS was defined as the time from diagnosis to death from any cause. Kaplan–Meier method was used for OS analysis and differences in survival were determined by Log-Rank. All statistical tests were two-sided and *P* values of <0.05 were considered to be significant. Statistical analysis was carried out using JMP 14 (SAS Institute, Cary, NC) statistical software.

Of the 829 patients, 148 (18%) had a VH NT-proBNP (≥8500 ng/L). Patients with VH NT-proBNP were older (67 versus 64 years, *P* = 0.004). Supplementary Table 1 shows the characteristics of the two patient subgroups. The VH NT-proBNP subgroup had higher baseline median levels of bilirubin (*P* < 0.001), alkaline phosphatase (*P* < 0.001) and dFLC (*P* < 0.001). For patients without VH NT-proBNP, the median urinary protein was significantly higher (*P* = 0.006), the serum creatinine was lower (*P* < 0.0001) and glomerular filtration rate (GFR) was higher (*P* < 0.001). Patients with VH NT-proBNP were older than patients with NT-proBNP <8500 ng/L. This may reflect delayed diagnosis in older patients, seeing that symptoms in amyloidosis are non-specific and are often attributed to comorbidities [11], which are more common in an elderly population.

**Table 1:** Early mortality based on NT-proBNP and stage.

			*N*	% Dead at			
				3 months	6 months	9 months	12 months
NT-proBNP ≥ 8500	All patients		148	38	58	68	74
	2012 Stage	I	0	–	–	–	–
		II	6	17	17	28	38
		III	40	27	45	56	61
		IV	102	45	66	76	82
	2004 Stage	I	0	–	–	–	–
		II	20	32	45	50	57
		IIIa	0	–	–	–	–
		IIIb	128	39	60	72	77
NT- proBNP < 8500	All patients		681	7	15	18	21
	2012 Stage	I	198	2	4	5	7
		II	193	4	8	13	14
		III	160	10	22	26	28
		IV	130	17	32	38	43
	2004 Stage	I	166	1	2	3	4
		II	309	6	14	18	19
		IIIa	206	13	26	33	37
		IIIb	0	–	–	–	–

*N* number, *NT-proBNP* N-terminal pro-brain natriuretic peptide.

**Fig. 1:**
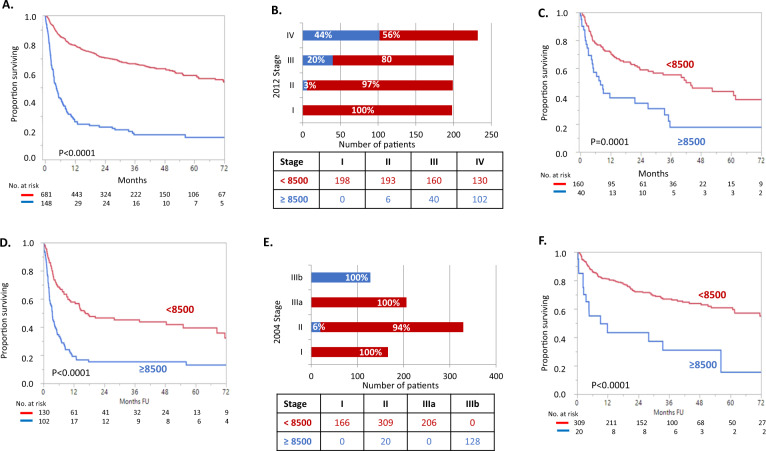
Interaction between biomarker stage and very high levels of NT-proBNP. **A** Survival parsed by NT proBNP ≥ 8500 pg/mL. **B** Proportion of patients surviving by Mayo 2012 stage parsed by NT-proBNP. **C** Overall survival of the Mayo 2012 Stage III with NT-proBNP parsed by NT-proBNP. **D** Overall survival of the Mayo 2012 Stage IV with NT-proBNP parsed by NT-proBNP. **E** Proportion of patients surviving by European modification of Mayo 2004 staging and parsed by NT-proBNP. **F** Overall survival of the Mayo 2004 Stage II patients parsed by NT-proBNP.

With a median follow-up of 30 months, median OS for VH NT-proBNP patients was 4.4 months in contrast to patients without VH NT-proBNP at 77.3 months (*P* < 0.0001) (Fig. 1A). Breakdown of early death for patients with VH NT-proBNP by stage is shown in Table 1. Seventy-four percent of the patients with VH NT-proBNP died in the first year. Of Mayo 2012 patients staged I, II, III, and IV, the percent of patients with VH NT-proBNP was 0%, 3%, 20%, and 44% (Fig. 1B). Patients with VH-NT-proBNP had early death rates comparable to (or even worse than) patients without VH NT-proBNP but who were a stage higher. For example, 3-month mortality for Mayo 2012 Stage III and VH NT-proBNP was 27% as compared to 17% among Stage IV without VH-NT-proBNP (Table 1; Fig. 1C, D). A similar pattern was seen for the European modification of the Mayo 2004 system though the addition of the VH NT-proBNP qualifier to the European modification of the Mayo 2004 staging system had less overall impact since it is already part of the definition (Table 1; Fig. 1E, F).

Thirty-nine patients (26%) in the VH NT-proBNP cohort lived greater than 1 year. Patients that survived 1 year or longer had lower levels of bilirubin (*P* = 0.005), uric acid (*P* = 0.0024), GFR levels (*P* = 0.04), and lower albumin (*P* = 0.02), but higher urinary protein (*P* = 0.02). These findings suggest that the VH NT-proBNP patients had delayed diagnosis and more extensive disease at diagnosis and that patients with renal presentations may have earlier diagnosis. Also of note, among the VH-NT-proBNP group there was no difference in OS between patients with low GFR and those with normal kidney function.

In terms of treatment, there were missing data about first-line therapy for 246 patients, 207 (31%) in the <8500 group and 39 (26%) in the VH-NT-proBNP group. Only 3% of the VH NT-proBNP patients received an ASCT in contrast to 29% of patients without VH NT-proBNP (*P* < 0.0001). The majority of patients received alkylators and bortezomib as the first-line treatment. Among the VH NT-pro BNP group, 76 patients received the combination of PI and an alkylator, 14 received PI only and 14 received alkylators only. Among patients that received therapy, there was no difference in bortezomib use between the 1-year survivors and non-survivors (81% versus 78%, *P* = 0.74). None of the five patients who received daratumumab-based therapy at the first line had a VH NT-proBNP. Forty patients, only 3 of whom had VH NT-proBNP, received daratumumab as part of second-line therapy.

Prospective data about daratumumab in patients with VH NT-proBNP are lacking, as they were excluded from the ANDROMEDA trial [7], The best data for daratumumab use in AL amyloidosis patients with VH NT-proBNP is a retrospective study from Heidelberg, which included 168 patients with relapsed disease treated with daratumumab and dexamethasone with (DVD) or without (DD) bortezomib [12]; 25 patients had NT-proBNP >8500 ng/L. For patients with VH NT-proBNP, the 1-year OS rates were 32% and 48% for DVD and DD treated patients; these results may well be superior to 23% survival rate seen in our study, in which none of the VH NT-proBNP patients were treated with daratumumab though our population is a newly diagnosed population, and theirs is a previously treated population. In the Heidelberg study, none of the patients stopped treatment with daratumumab because of toxicity. making it a potentially appealing treatment option for study among patients with VH NT-proBNP.

Although other retrospective studies have evaluated “ultra-high risk” amyloidosis [13–15], focusing on the cardiac stage, we approached the problem from a pragmatic standpoint. Our goal was to define those populations who are typically included and excluded from clinical trials, i.e., using the NT-proBNP threshold of 8500 ng/L as defined by Wechalekar et al. [4]. We found that 18% of newly diagnosed AL amyloidosis patients seen at the Mayo Clinic had an NT-proBNP ≥8500 ng/L and consistent with other reports had a poor OS with 74% dead in the first year. Importantly, survival by stage is considerably better once the VH NT-proBNP patients are removed. This has important implications for trial design using this important variable.

This study is unique in that it compares a cohort of patients with VH NT-proBNP to patients without VH NT-proBNP levels, and this comparison yielded several interesting observations, but it does have several limitations. It is a retrospective single-center study performed at a tertiary center, and the induction regimens were not uniform. Despite these limitations, the data are important since they can serve as a potential benchmark for expected outcomes both among the sickest AL amyloidosis patients and among patient groups from which the sickest patients have been excluded. Patients with VH NT-proBNP should be considered for specially designed trials, in pursuit of personalized treatment.

### Supplementary information

Table 1 sup baseline patient characteristics
