# Correction: Outcomes among newly diagnosed AL amyloidosis patients with a very high NT-proBNP: implications for trial design

**DOI:** 10.1038/s41375-024-02311-w

**Published:** 2024-06-17

**Authors:** I. Vaxman, S. K. Kumar, F. Buadi, M. Q. Lacy, D. Dingli, Y. Hwa, A. Fonder, M. Hobbs, S. Hayman, T. Kourelis, R. Warsame, E. Muchtar, N. Leung, P. Kapoor, M. Grogan, R. Go, Y. Lin, W. Gonsalves, M. Siddiqui, R. A. Kyle, S. V. Rajkumar, M. A. Gertz, A. Dispenzieri

**Affiliations:** 1 Division of Hematology, Mayo Clinic, Rochester, MN USA; 2 Institute of Hematology, Davidoff Cancer Center, Rabin Medical Center, Petah Tikva, Israel; 3 Sackler Faculty of Medicine, Tel-Aviv University, Tel-Aviv, Israel; 4 Department of Cardiovascular Medicine, Mayo Clinic, Rochester, MN USA

**Keywords:** Risk factors, Myeloma

Correction to: *Leukemia* 10.1038/s41375-021-01297-z, published online 21 May 2021

The Funding information section was missing from this article and should have read ‘This work was supported by the Paul Calabresi K12 Career Development Award (CA90628-21) and in part by Grant Number P30 CA015083 and CA186781 from the National Cancer Institute and by the Predolin Foundation and the JABBS Foundation.’.

The original article has been corrected.

