# Supplementary material for: Outcomes among newly diagnosed AL amyloidosis patients with a very high NT-proBNP: implications for trial design
Source: Leukemia. 2021 May 21;35(12):3604–7. doi: 10.1038/s41375-021-01297-z (PMC8632659; doi:10.1038/s41375-021-01297-z)
Supplement: Supplementary file 1 — Table 1 sup baseline patient characteristics [file 41375_2021_1297_MOESM1_ESM.docx]

Table 1: **Patient baseline characteristics and treatment**

| **Variable** | **NT-proBNP<8500 ng/L (N=681)** | **NT-proBNP≥8500 ng/L (N=148)** | **P value** |
| --- | --- | --- | --- |
| Age (years), median (range) | 64 (29-88) | 67 (38-89) | **0.004** |
| Male, n (%) | 97 (66) | 447 (66) | 0.98 |
| Organ involved, n (%) |  |  |  |
| Cardiac | 312 (46) | 148 (100) | **0.01** |
| Renal | 343 (50) | 55 (37) | 1.0 |
| GI | 147 (22) | 28 (19) | 0.62 |
| Liver | 73 (11) | 12 (8) | 0.57 |
| Autonomic nerve | 84 (12) | 14 (9) | 0.63 |
| NT-pro BNP, ng/L | 1393 (331-3466) | 14288 (10965-21142) | **<0.0001** |
| Serum ALP (U/L) | 83 (66-115) | 105 (75-143) | **<0.0001** |
| Serum bilirubin (umol/L) | 0.5 (0.3-0.7) | 0.7 (0.5-1.2) | **<0.0001** |
| GFR (mL/min). | 65 (48-83) | 44 (28-62) | **<0.0001** |
| Serum creatinine | 1.05 (0.8-1.4) | 1.5 (1.1-2.2) | **<0.0001** |
| Proteinuria (g/24h) | 1.1 (0.2-4.4) | 0.5 (0.2-2) | **0.006** |
| Serum albumin, g/L | 3.0 (2.4-3.4) | 3.1 (2.6-3.3) | 0.48 |
| Involved light chain |  |  | 0.098 |
| Kappa, mg/dL n (%) | 112 (20) | 17 (13) |  |
| Lambda, mg/dL, n (%) | 342 (59) | 85 (67) |  |
| dFLC mg/dL, mean (IQR) | 77 (38-117) | 101 (75-128) | **<0.0001** |
| BMPC at diagnosis, median (IQR) | 14 (13-15) | 17(14-20) | 0.057 |
| First line treatment^†, ‡^ |  |  |  |
| ASCT, n (%) | 195 (29) | 5 (3) | **<0.0001** |
| Bortezomib, n (%) | 413 (61) | 90 (61) | 0.97 |
| IMiD, n (%) | 36 (5) | 3 (2) | 0.13 |
| Daratumumab, n (%) | 5 (1) | 0 (0) | 0.6 |
| Alkylators, n (%) | 408 (60) | 90 (61) | 0.85 |
| Second line treatment^†^ |  |  |  |
| Bortezomib, n (%) | 100 (15) | 13 (8) | 0.065 |
| IMiD, n (%) | 38 (6) | 5 (3) | 0.4 |
| Daratumumab, n (%) | 37 (5) | 3 (2) | 0.09 |
| Alkylators, n (%) | 67 (10) | 8 (5) | 0.11 |

* Unless otherwise stated, results are represented as median (interquartile range)

†All patients who received treatment received dexamethasone as part of their therapy

^‡^There was missing data about first line therapy for 246 patients, 207 (31%) in the <8500 group and 39 (26%) in the VH-NT-proBNP group

N=number; Gi=gastrointestinal; ALP=alkaline phosphatase; GFR=glomerular filtration rate; ASCT=autologous stem cell transplantation; IMiD=immunomodulatory agent; NT-proBNP= N-terminal pro-brain natriuretic peptide
